# Supplementary material for: Clear Native Gel Electrophoresis for the Purification of Fluorescently Labeled Membrane Proteins in Native Nanodiscs
Source: Anal Chem. 2025 Aug 1;97(31):16796–804. doi: 10.1021/acs.analchem.5c01702 (PMC12355471; doi:10.1021/acs.analchem.5c01702)
Supplement: Supplementary file 1 [file ac5c01702_si_001.pdf]

Supplementary Information

Clear Native Gel Electrophoresis for the Purification of Fluorescently Labeled Membrane Proteins in Native Nanodiscs

Bence Ezsias[1], Nikolaus Goessweiner-Mohr[1], Christine Siligan[1], Andreas Horner[1], Carolyn Vargas[2,3,4], Sandro Keller[2,3,4], Peter Pohl[1,*]

[1]Institute of Biophysics, Johannes Kepler University Linz, Gruberstraße 40, Linz 4020 Austria, [2]Biophysics, Institute of Molecular Biosciences (IMB), NAWI Graz, University of Graz, Humboldtstr. 50/III, 8010 Graz, Austria, [3]Field of Excellence BioHealth, University of Graz, 8010 Graz, Austria, [4]BioTechMed-Graz, 8010 Graz, Austria

Content:

Figure S1. Autocorrelation functions obtained from fluorescence correlation spectroscopy (FCS) measurements of 1 nM Alexa Fluor 647 maleimide in solution. S2

Table S1. Molecular brightness and diffusion residence times within the confocal volume**, derived** from FCS measurements of SEC-purified samples **of** Alexa Fluor 647–labeled proteins**.** S2

Calibration procedure. Approach used to determine confocal radii and effective volumes for FCS analysis. S3

Table S2. Confocal radii and effective volumes determined at the time of the respective protein measurements. S3

Figure S2. High-resolution clear native PAGE (CNE), size-exclusion chromatography (SEC), and fluorescence correlation spectroscopy (FCS) analysis of the voltage-gated potassium channel KvAP. S4

Figure S3. Detergent-free clear native PAGE (CNE), size-exclusion chromatography (SEC) and fluorescence correlation spectroscopy (FCS) of the voltage-gated potassium channel KvAP. S5

Approach for the estimation of particle concentration, protein and nanodisc areas S6

Table S3. Estimated areas of the monomeric or tetrameric GlpF and the purified nanodiscs and the estimation of the average number of lipids per nanodisc. S6

Reference S6

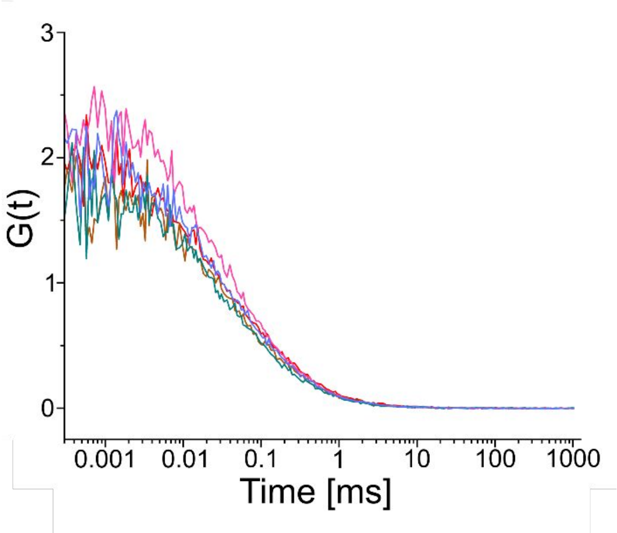

Figure S1. Autocorrelation functions obtained from fluorescence correlation spectroscopy (FCS) measurements of 1 nM Alexa Fluor 647 maleimide in solution. These control measurements were performed prior to each protein measurement to determine molecular brightness as well as confocal radius and volume. The traces correspond to measurements taken before GlpF/blue native PAGE (red), NavMs/high-resolution CNE (magenta), KvAP/high-resolution CNE (brown), KvAP/detergent-free CNE (cyan), and HpUreI/detergent-free CNE (purple). A 3D diffusion model with an extended triplet state component was used to fit the autocorrelation curves.

	Molecular brightness [kHz]	Residence time [µs]
GlpF/BNE	7.5	120
NavMs/hrCNE	6.8	85
KvAP/hrCNE	5.9	80
KvAP/CNE	6	89
HpUreI/CNE	5.8	99

Table S1. Molecular brightness and diffusion residence times within the confocal volume, derived from FCS measurements of SEC-purified samples of the indicated proteins labeled of Alexa Fluor 647labeled proteins.

Calibration procedure. Approach used to determine confocal radii and effective volumes for FCS analysis.

The confocal radius is estimated from the measured residence time and the literature value of the diffusion coefficient of the Alexa Fluor 647 dye (Equation S1):

$$r_0^2 = D * 4 * \tau \qquad \text{(S1)}$$

where r_0 is the confocal radius, D is the diffusion coefficient and τ is the residence time.

The effective volume is then estimated [1] (Equation S2):

$$V_{eff} = \pi^{3/2} * r_0^3 * \kappa \qquad \text{(S2)}$$

where V_{eff} is the effective volume and κ is the structural parameter (~8 for 60x objective, 633 laser), which describes the shape of the effective volume.

	Confocal radius [nm]	Effective volume [fL]
GlpF/BNE	398	2.81
NavMs/hrCNE	335	1.67
KvAP/hrCNE	325	1.53
KvAP/CNE	343	1.79
HpUreI/CNE	361	2.10

Table S2**.** Confocal radii and effective volumes determined at the time of the respective protein measurements.

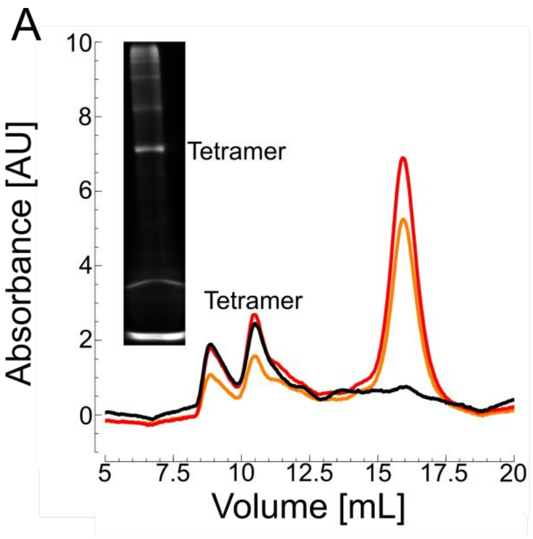

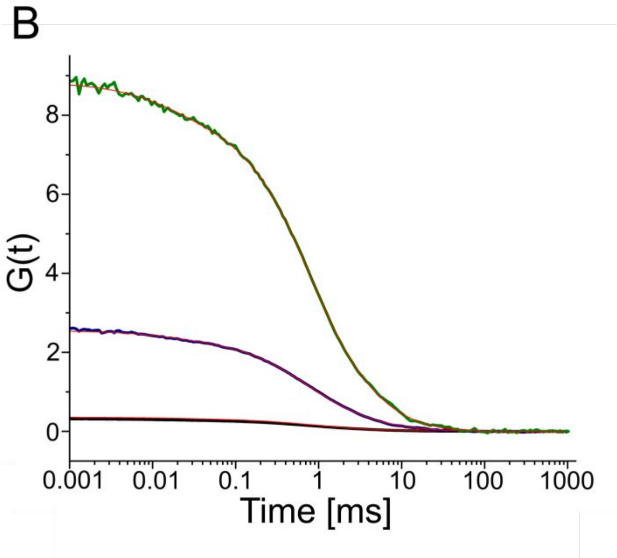

***Figure S2. High-resolution clear native PAGE (CNE), size-exclusion chromatography (SEC) and fluorescence correlation spectroscopy (FCS) analysis of the voltage-gated potassium channel* KvAP**. *A*: SEC and high-resolution CNE fluorescent image of KvAP; SEC shows three major peaks, one indicating aggregate (at 8.5–9 mL), one for a tetramer (at 10–11 mL) and one for smaller species (at 15–16 mL). The black curve is the absorbance at 280 nm, the red at 650 nm, and the orange at 665 nm. *B*: Autocorrelation functions of tetrameric KvAP measured after SEC (black); after extraction from the total protein fraction and separation by high-resolution CNE (blue); or after extraction from SEC-purified fractions and separation by high-resolution CNE (green). The measured sample concentrations were 2.7°nM, 0.3°nM and 0.1°nM, respectively. The diffusion time and molecular brightness before CNE were 880±64.1 µs and 44±0.5 kHz, respectively. After CNE, these values were 770±107 µs and 48±1.7 kHz, respectively.

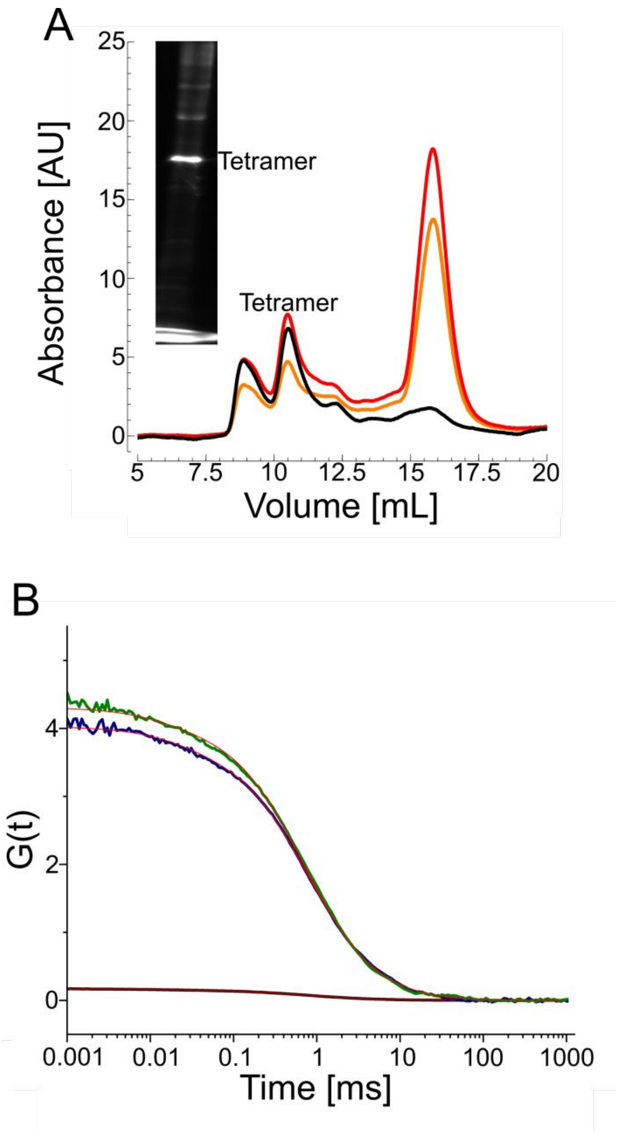

***Figure S3. Detergent-free clear native PAGE (CNE), size-exclusion chromatography (SEC) and fluorescence correlation spectroscopy (FCS) of the voltage-gated potassium channel* KvAP**. *A*: SEC and detergent-free CNE fluorescent image of KvAP; SEC shows three major peaks, indicating aggregates (at 9 mL), tetramers (at 10–11 mL), and a smaller species (at 15–16 mL). The black curve is the absorbance at 280 nm, red at 650 nm, and the orange at 665 nm. *B*: Autocorrelation functions of tetrameric KvAP measured after SEC (black); after extraction from the total protein fraction and separation by detergent-free CNE (blue); or after extraction from SEC-purified fractions and separation by detergent-free CNE (green). The measured sample concentrations were 7°nM, 0.27°nM and 0.25°nM, respectively. The diffusion time and molecular brightness before CNE were 747±52.6 µs and 43±1.8 kHz, respectively. After CNE, these values were 760±20 µs and 38±1.5 kHz, respectively. The concentration of the extracted tetramer was 0.27 nM in 300 µL standard buffer.

Estimates of particle concentration, protein and nanodisc areas

The particle concentration is estimated using the following equation (Equation S3):

$$C = \frac{N}{V_{eff} * N\mathrm{A}} \quad \text{(S3)}$$

where C is the concentration, V_{eff} is the effective confocal volume, N_{A} is Avogadro's constant, and N is the number of particles within V_{eff}. N is derived from the amplitude of the autocorrelation curve and is inversely proportional to $G(0)$.

The average number of lipids per nanodisc is estimated by subtracting the diameter of the membrane protein (determined in PyMOL) from the total diameter of the nanodisc. The resulting lipid annulus area is then divided by the cross-sectional area of a single lipid head group, assumed to be approximately 0.7 nm².

Form	A_{protein} [nm^2]	A_{nanodisc} [nm^2]	$N_{\mathrm{lipid/nanodisc}}$
Monomer	12.6	30	49
Tetramer	49	174	357

Table S3. Estimated areas of the monomeric or tetrameric GlpF and the purified nanodiscs and the estimation of the average number of lipids per nanodisc.

Protein	A_{oligomer} [nm^2]	A_{nanodisc} [nm^2]	$N_{\mathrm{lipid/nanodisc}}$
GlpF	49	174	357
NavMs	28.3	222	635
KvAP (hrCNE)	86.6	306	628
KvAP (CNE)	86.6	240	440
HpUreI	67.9	223	444

Table S4. Estimated areas of oligomers and the purified nanodiscs and the estimation of the average number of lipids per nanodisc.

Reference

1 Ries, J. & Schwille, P. Studying slow membrane dynamics with continuous wave scanning fluorescence correlation spectroscopy. *Biophys. J.* **91**, 1915-1924, doi:10.1529/biophysj.106.082297 (2006).
